# Kidney trajectory charts improve GP management of patients with reduced kidney function: a randomised controlled vignette study

**DOI:** 10.3399/BJGPO.2023.0193

**Published:** 2024-04-03

**Authors:** Michelle Guppy, Paul Glasziou, Mark Jones, Elaine Beller, Jonathan E Shaw, Elizabeth Barr, Jenny Doust

**Affiliations:** 1 Institute for Evidence-Based Healthcare, Faculty of Health Sciences and Medicine, Bond University, Gold Coast, Australia; 2 Baker Heart and Diabetes Institute, Melbourne, Australia; 3 Menzies School of Health Research, Charles Darwin University, Darwin, Australia; 4 Australian Women and Girls’ Health Research (AWaGHR) Centre, School of Public Health, Faculty of Medicine, The University of Queensland, Herston, Australia

**Keywords:** aging, Australia, general practice, glomerular filtration rate, kidney, overdiagnosis, primary health care, renal insufficiency, chronic

## Abstract

**Background:**

The stages of chronic kidney disease (CKD) and estimated glomerular filtration rate (eGFR) reference ranges are currently determined without considering age.

**Aim:**

To determine whether a chart that graphs age with eGFR helps GPs make better decisions about managing patients with declining eGFR.

**Design & setting:**

A randomised controlled vignette study among Australian GPs using a percentile chart plotting the trajectory of eGFR by age.

**Method:**

Three hundred and seventy-three GPs received two case studies of patients with declining renal function. They were randomised to receive the cases with the chart or without the chart, and asked a series of questions about how they would manage the cases.

**Results:**

In an older female patient with stable but reduced kidney function, use of the chart was associated with GPs in the study recommending a longer follow-up period, and longer time until repeat pathology testing. In a younger male First Nations patient with normal but decreasing kidney function, use of the chart was associated with GPs in the study recommending a shorter follow-up period, shorter time to repeat pathology testing, increased management of blood pressure and lifestyle, and avoidance of nephrotoxic medications. This represents more appropriate care in both cases.

**Conclusion:**

Having access to a chart of percentile eGFR by age was associated with more appropriate management review periods of patients with reduced kidney function, either by greater compliance with current guidelines or greater awareness of a clinically relevant kidney problem.

## How this fits in

Age is not currently considered as a factor in the definition of patients with chronic kidney disease, and current guideline recommendations do not take age into account. This research shows that considering age with a kidney trajectory chart can improve GP management recommendations for patients with declining renal function. A chart that plots eGFR with age may be a useful clinical tool to assist clinicians in their management of patients with declining renal function.

## Introduction

Chronic kidney disease (CKD) is currently defined as a glomerular filtration rate (GFR) below 60 mL/min/1.73 m^2^ or GFR >60 mL/min/1.73 m^2^, with persistent albuminuria or other markers of kidney damage for at least 3 months.^1^ Age is not considered as a factor in the stages of CKD, and GFR reference ranges are currently not age dependent,^1^ despite the fact that kidney function declines with age as a natural process in the absence of disease.^2,3^ There is debate about whether age should be considered, given that 40% of people aged over 70 years have a GFR that meets the definition of CKD,^4^ and whether the early stages of CKD (particularly 3a and 3b) should be considered diseases.^4,5^ There have been recommendations to lower the CKD threshold in people aged over 65 years to a GFR of <45 mL/min/1.73 m^2^;^6,7^ however, current guidelines have not changed to incorporate this recommendation,^8–10^ the rationale being that older populations are still at risk of adverse outcomes with higher CKD stages.^9^ In the higher stages of CKD, guidelines recommend monitoring and medication to reduce cardiovascular risk.^1,8^ However, it can be difficult to explain to patients what CKD means, and when is appropriate to refer to a nephrologist.^5^

In younger people with a declining GFR, kidney disease may be underrecognised if their GFR remains in the normal range. Younger adults can have adverse clinical outcomes with a modest reduction in GFR in the higher ranges.^11^ There have been recommendations to change the threshold GFR to define CKD in younger people using percentiles or age-adapted staging, to recognise a clinically important decline at higher GFR values.^12^ The current mode of reporting without an age reference may lead to underrecognition of a clinically significant decline in younger people.^11^

There have been calls for the use of the concept of ‘kidney age’ when GPs make decisions for patients about declining estimated glomerular filtration rate (eGFR).^13^ The UK NICE Clinical Knowledge Summaries consider age as well as baseline GFR when giving clinical examples for the application of the guidelines.^14^ There have also been recommendations to use a kidney percentile chart when considering kidney function values.^12^ We sought to investigate this concept by developing a percentile chart of kidney function with age (Supplementary File), and then testing it with GPs to see how they would use it to manage two hypothetical patient cases (clinical vignettes).^15^ The concept of the kidney age trajectory chart, how it was developed, and tests of its effect on GP diagnosis of CKD has been previously described, and showed the utility of the chart in helping GPs to recognise the clinical significance of eGFR results.^16^ For the current study, we posed the question, ‘Does a kidney age trajectory chart improve GP management of patients with declining kidney function?’ We were interested in determining whether a chart helps GPs to recognise whether a patient’s kidney function is similar to patients of the same age and sex, and if this would help GPs make better decisions about managing those patients. This article describes the effect of the chart on GPs’ proposed management of the two patient cases.

## Method

### Participants

An electronic questionnaire using Qualtrics XM was sent by a third party database (AMPCo) to a stratified random sample of 9500 Australian GPs asking them to participate in the study. The randomised vignette study presented two patient case studies, and a percentile chart of kidney function with age.

The study took place between August 2018 and November 2018. GPs were randomised in a 1:1 ratio by the Qualtrics XM program to receive the case studies with the chart (immediate chart group), or without the chart (delayed chart group), and were asked a series of questions about how they would manage the cases. GPs who did not receive the chart immediately were subsequently given the chart and asked the same questions a second time. GPs with access to the chart were first asked to find the patient’s percentile kidney function on the chart before responding to the questions. The order of cases was allocated randomly.

### Case vignettes

Case 1 was a 76 year old woman (no ethnicity specified) who had an eGFR in the CKD stage 3a range, which had been stable over a 12 months. Her eGFR was 58 mL/min/1.73 m^2^ (just below the 50th percentile for her age) and she had no albuminuria. The woman was otherwise well and healthy, although she was overweight and had a slightly elevated blood pressure (140/90 mmHg). Her lipid levels (and reference ranges in mmol/L) were: total cholesterol (TC) 5.4 (3.9–5.5), low-density lipoprotein (LDL) 3.3 (0–4), high-density lipoprotein (HDL) 1.81 (1.1–1.9), and fasting glucose 5.0 (3–6). Using the current Australian risk calculator, the woman’s absolute risk of cardiovascular disease in the next 5 years was 5%, below the threshold currently recommended for lipid-lowering therapy, although this information was not explicitly given in the vignette.^16^

Case 2 was a 45 year old First Nations man with an eGFR that was in the normal range, but had decreased by 5 mL/min/1.73 m^2^ over the preceding 12 months. His eGFR was 65 mL/min/1.73 m^2^ (5th percentile for his age) with no albuminuria. The man was otherwise well and healthy, although he was overweight and had a slightly elevated blood pressure (140/90 mmHg). He also used to smoke (had quit 5 years previously), and had a strong family history of diabetes. His lipid levels (and reference ranges in mmol/L) were: total cholesterol 5.4 (3.9–5.5), LDL 3.3 (0–4), HDL 1.81 (1.1–1.9), and fasting glucose 5.0 (3–6). His estimated risk of cardiovascular disease in the next 5 years was 2%, although it is recognised that the risk calculator will underestimate risk in First Nations people.^16^

For each case, participants were asked:

- Question 1. When would you review the patient? (Options: 1 week, 3 months, 6 months, 12 months, only as required).
- Question 2. When would you repeat the patient’s pathology tests? (Options as above).
- Question 3. What is your management plan for this patient? (Options: no specific management, blood pressure reduction, lipid-lowering treatment, lifestyle modification for weight management, avoidance of nephrotoxic medications, referral to a nephrologist for advice. More than one option could be selected).

### Statistical analysis

Our primary comparison was between GPs who had access to the chart with the case (immediate chart group) and GPs who only had the cases (delayed chart group). A secondary comparison was made between the responses of the GPs in the delayed chart group before and after seeing the chart.

Analysis was performed using SAS (version 3.81). Multinomial logistic regression was used to estimate odds ratios, 95% confidence intervals, and *P* values, for comparing the distribution of the five timing categories (as stated above) chosen for the next review and pathology testing between treatment groups. The reference group was 12 months for the older woman and 3 months for the younger First Nations man. Binary logistic regression was used to estimate odds ratios, 95% confidence intervals, and *P* values for the comparison of probability of choosing each of the six individual management options (as stated above) between treatment groups. For the within-participants comparisons involving the delayed chart group only, McNemar’s test was used to compare the six management options before and after observing the chart. Bowker’s test of symmetry was used to compare the timing of the next review and pathology testing before and after observing the chart.

## Results

**Figure 1.**
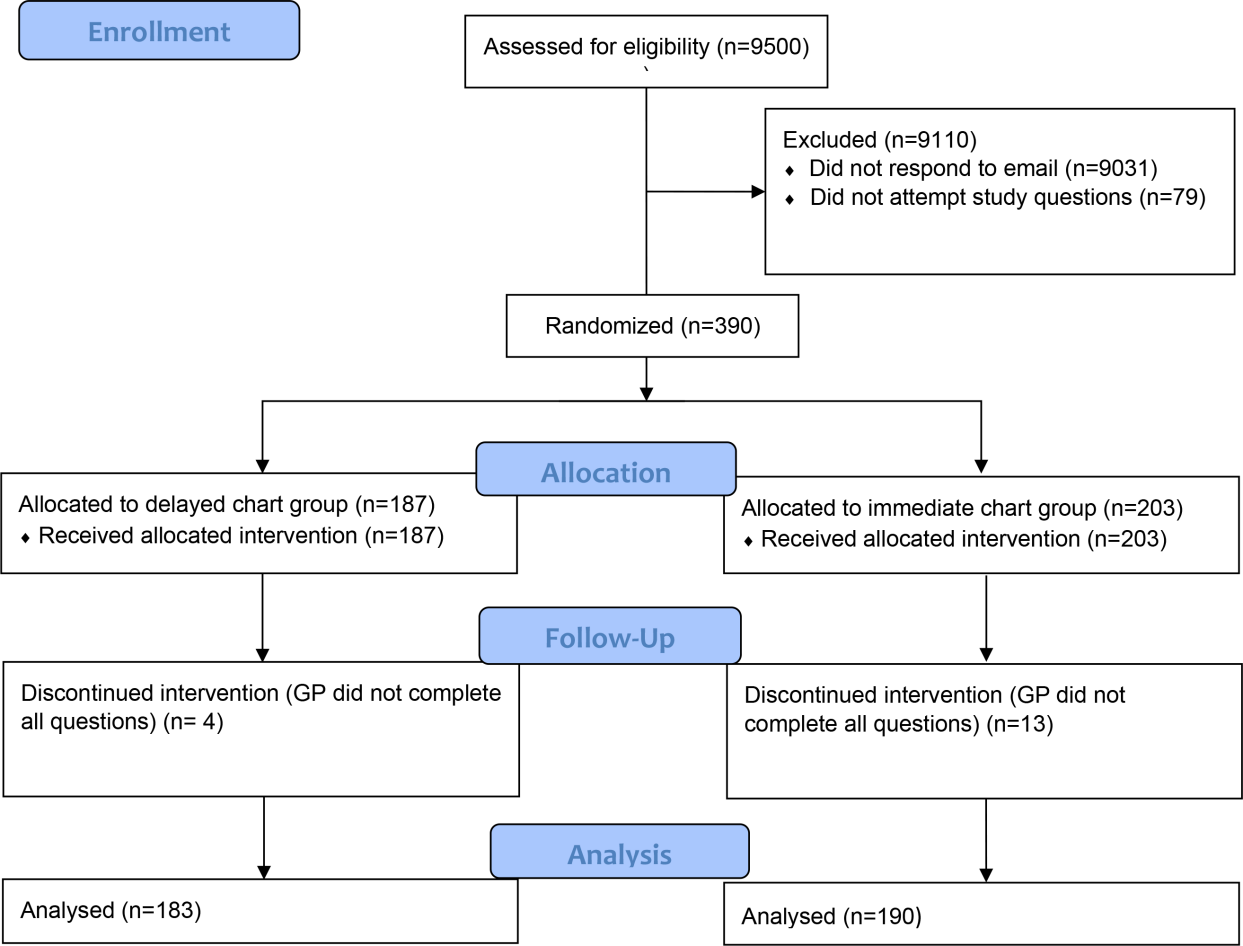
CONSORT flow diagram

We received 496 responses to the email invitation; of these responders, 390 participated in the randomised vignette study. Seventeen GPs did not complete all questions in the case studies, and so were excluded from the analysis after randomisation. A total of 373 responses were analysed (190 in the immediate chart group and 183 in the delayed chart group) (Figure 1). See Table 1 for participant demographics.

**Table 1.** Participant demographics

	Delayed chart(*n* = 183)^a^					Immediate chart(*n* = 190)		
	*n*			%		*n*		%
**Sex**								
Female	95			52.5		93		48.9
Male	86			47.5		97		51.1
**Age**								
<20–29 years	6			3.3		9		4.7
30–39 years	46			25.4		56		29.5
40–49 years	50			27.6		41		21.6
50–59 years	39			21.5		41		21.6
60–69 years	29			16.0		35		18.4
70–79 years	11			6.1		8		4.2
**Years of experience**								
GP registrar	12			6.7		27		14.2
<5 years	24			13.3		30		15.8
5–10 years	39			21.7		30		15.8
11–20 years	36			20.0		32		16.8
21–30 years	30			16.7		30		15.8
>30 years	39			21.7		41		21.6

^a^Some included participants did not specify all demographic values.

### Primary analysis

#### Case 1: older woman

**Figure 2.**
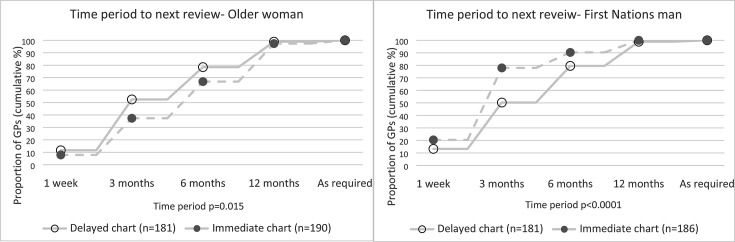
(**A**) (Case of older woman) and (**B**) (Case of First Nations man). Time period chosen by the GPs (cumulative %) for next review of the older woman and First Nations man before viewing the chart (delayed chart group) compared with viewing the chart at the time of seeing the case vignette (immediate chart group). Connecting lines highlight difference in cumulative proportion between groups.

GPs in the immediate chart group chose longer intervals to review the patient than GPs in the delayed chart group. In the delayed chart group, 78% proposed that they would see her within 1 week to 6 months, compared with 67% who had the chart immediately. In the delayed chart group, 22% would review her at 12 months, compared with 33% with the chart immediately (*P* = 0.015) (Figure 2a). Similar group differences were also reflected in the proposed time to repeat pathology testing. GPs in the immediate chart group were more likely to propose repeat pathology testing at 12 months compared with GPs in the delayed chart group (41% vs 29%, *P* = 0.012).

**Figure 3.**
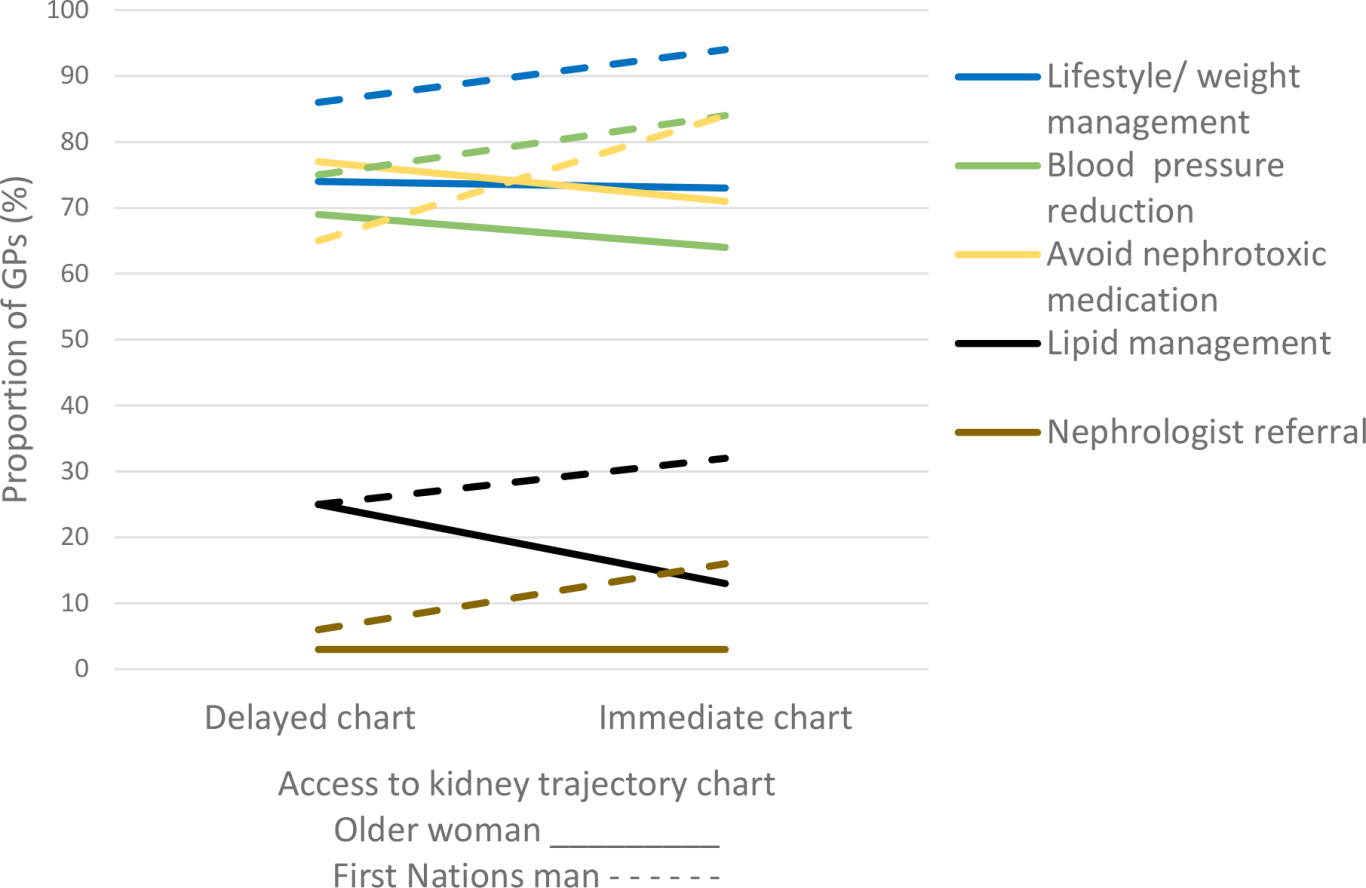
GP management decisions for the cases of the older woman and First Nations man before viewing the chart (delayed chart group) compared with viewing the chart at the time of seeing the case vignette (immediate chart group).

The majority of GPs in both the immediate and delayed chart groups would treat the older woman’s blood pressure, advocate for lifestyle advice, avoid prescribing nephrotoxic medications, and continue to manage the patient themselves (Figure 3, Table 2). Access to the chart reduced the likelihood that GPs would prescribe lipid-lowering therapy in the older woman (*P* = 0.003) (Figure 3, Table 2).

**Table 2.** Proportion (%) of GPs without the chart (delayed = reference group) compared with GPs with the chart (immediate) who would recommend each management option

	Older woman			First Nations man		
**Management option**	Delayed	Immediate	OR (95% CI)	Delayed	Immediate	OR (95% CI)
Recommended	*n* = 183	*n* = 190		*n* = 182	*n* = 190	
Blood pressure reduction	69%	64%	0.80(0.52 to 1.20)	75%	84%	1.70(1.00 to 2.80)
Lipid management	25%	13%	0.44(0.26 to 0.76)	25%	32%	1.40(0.89 to 2.20)
Lifestyle/weight management	74%	73%	0.92(0.58 to 1.47)	86%	94%	2.60(1.20 to 5.40)
Avoid nephrotoxic medication	77%	71%	0.71(0.45 to 1.20)	65%	84%	2.80(1.70 to 4.50)
Nephrologist referral	3%	3%	1.20(0.35 to 3.90)	6%	16%	3.40(1.60 to 7.10)

CI = confidence interval. OR = odds ratio.

#### Case 2: First Nations man

GPs in the immediate chart group were more likely to choose earlier time intervals to review the First Nations man compared with the delayed chart group. Seventy-eight per cent of GPs with the chart immediately would review him in 3 months or sooner, compared with only 50% in the delayed chart group (*P*<0.0001). (Figure 2b) Sixty per cent of GPs in the immediate chart group also chose to repeat pathology testing in 3 months or sooner, compared with 39% in the delayed chart group (*P*<0.0001).

GPs in the immediate chart group were more likely to recommend blood pressure reduction for this patient case (*P* = 0.046), more likely to recommend lifestyle intervention (*P* = 0.012), and more likely to avoid nephrotoxic medications (*P*<0.0001) compared with the delayed chart group (Table 2, Figure 3).

Most GPs would not prescribe lipid-lowering treatment for the First Nations man, and there was no difference between the groups (immediate and delayed chart) (*P* = 0.15). Most GPs would not refer the First Nations man to a nephrologist, but access to the chart was associated with an increase in the proportion of GPs wanting to seek further advice (6% delayed chart vs 16% immediate chart, *P* = 0.0015), (Table 2, Figure 3).

In the case of the older woman, after seeing the chart, GPs became more likely to choose to review the patient at a longer time period (*P* = 0.014) and repeat her pathology testing at a longer time period (*P* = 0.012). They were also more likely to not treat the older woman’s blood pressure (38%) compared with before they had seen the chart (31%, *P* = 0.005). Before seeing the chart, the vast majority would not refer to a nephrologist (97%). However, after receiving the chart, an additional small minority (4%) of GPs would consider referring to a nephrologist for advice (*P* = 0.04). Otherwise, access to the chart had no influence on whether the GPs would prescribe lipid-lowering therapy, advise lifestyle management, or avoid nephrotoxic medications for this case.

With the First Nations male case, after seeing the chart, GPs were more likely to choose to review this patient in a shorter time period, compared with before they had the chart (*P*<0.0001) and to repeat the pathology tests sooner (*P*<0.0001). After seeing the chart, GPs were more likely to recommend blood pressure reduction therapy (87% versus 75%, *P* = 0.0003), more likely to commence lipid-lowering therapy (45% versus 25%, *P*<0.0001), more likely to avoid nephrotoxic medications (87% versus 65%, *P*<0.0001) and more likely to refer the First Nations man to a nephrologist (31% versus 5%, *P*<0.0001). Lifestyle management was unchanged by the chart, with the majority recommending this before and after the chart (*P* = 1.0).

## Discussion

### Summary

This randomised case vignette study showed that having access to a chart that graphs percentile kidney function (eGFR) by age was associated with more appropriate management review periods for the patient cases with reduced kidney function. In the case of the older woman with stable but reduced kidney function, use of the chart was associated with proposals for a longer follow-up period, longer time until repeat pathology testing, and reduced use of lipid-lowering medication. In the younger male First Nations patient, use of the chart was associated with proposals for a shorter follow-up period, shorter time to repeat pathology testing, increased management of blood pressure and lifestyle, and avoidance of nephrotoxic medications. Use of the chart reduced proposals for unnecessary pathology testing and unnecessary early follow-up.

### Strengths and limitations

This study was a case vignette study,^15^ with cases created for specific age, sex, ethnicity, biometric, and blood test values. The patient cases had borderline hypertension and lipid values for which GPs might have differences in management based on a patient-centred approach. Therefore, results may not reflect how a GP would actually manage these cases in a real clinical setting. We did not ask the participating GPs any questions regarding their reasons for changing their proposed management, which should be further explored. The chart used in this study was based on cross-sectional data from an Australian population survey.^17^ This may limit the generalisability of this chart to other population groups.

### Comparison with existing literature

CKD guidelines do not currently use age as part of the treatment recommendation algorithm. The guidelines state, *‘An eGFR of <60 mL/min/1.73 m* ^*2*^ *is common in older people but is nevertheless predictive of significantly increased risks of adverse clinical outcomes and should not be considered physiological or age-appropriate’*.^8^ There has been much debate about this statement, with reasons including that absolute cardiovascular risk increases with age, and therefore, CKD diagnosis is an important requirement to manage cardiovascular disease risk regardless of age.^9,18^ People with a moderate or severe reduction in eGFR (<45 mL/min/1.73 m^2^) (stage 4–5) are at the highest risk of cardiovascular disease.^18^ However, there is still debate about older people in stage 3a as to how aggressively these patients need to be managed. An individual approach is required with older people, taking into account their comorbidities, functional states, and personal priorities.^8,19^ A kidney trajectory chart can be a useful tool for GPs to help in a discussion with patients about their own functional status, likelihood of further decline in kidney function, and other health priorities.^12^

The older woman case scenario had stable kidney function in the CKD stage 3a category. According to the CKD guidelines,^8^ management should include follow-up every 12 months, with clinical assessment of blood pressure and weight, HbA1c, and fasting lipids annually, as well as assessing absolute cardiovascular risk and avoiding nephrotoxic medications.^8^ Without the chart, GPs were more likely to review this woman earlier than 12 months. Only one-quarter of GPs would prescribe lipid-lowering therapy for the older woman, and GPs with the chart were less likely to prescribe this (13%). We did not give the GP participants the patient’s complete lipid values, only TC, HDL, and LDL. CKD guidelines recommend use of statin therapy in older people with CKD regardless of their lipid levels.^8–10^ This woman had a 5-year absolute cardiovascular risk of 5%, putting her in the low-borderline-intermediate cardiovascular risk category, depending on which guidelines and risk calculator is used.^18,20,21^ We did not ask GPs any questions about why they changed their management plan for this patient. Reasons for this would be worth exploring, as there are some variations between guidelines, and recent changes to guidelines since this study was performed.^10,18,20,22^

Regarding the younger male case, his kidney function did not meet the definition of CKD according to the current guidelines.^8^ However, with a loss of 5 mL/min/1.73 m^2^ in the previous year, there could be cause for concern that requires a shorter follow-up period, given that a rapid loss of GFR in the normal range can be associated with an increased risk for end stage kidney disease, and increased cardiovascular risk. A recent study showed that among young people, a modest reduction in kidney function at a higher baseline GFR is associated with adverse cardiovascular and renal outcomes, and that these patients are often not picked up in the clinical guidelines because they fall outside the current definitions of CKD.^11,23^ For the younger male patient, recognition that his eGFR was in the 5th percentile for age was associated in our study with GPs choosing to repeat his tests earlier. Only with the age comparison chart was it clearly shown that his eGFR might be problematic.

### Implications for research and practice

A trial of use of this chart in clinical practice would be important to see whether it improves the recognition and management of reduced kidney function overall, and reduces overdiagnosis and concern in older people in the clinical environment. Our hypothesis is that the chart improves GP patient management of CKD, improves timeliness of review, and leads to more appropriate referral to nephrologists for advice. Further qualitative research exploring GPs’ reasons for choosing management options would be useful to further understand how the chart might work in clinical practice. Charts based on longitudinal data from broader population groups relevant to the clinical population of interest should be considered and trialled.

Having access to a percentile chart that graphs kidney function with age was associated with more appropriate management review periods for patient case scenarios with reduced kidney function, either by greater compliance with current guidelines, or greater awareness of a clinically relevant problem. A trial of use of this chart in clinical practice would be useful to see whether it improves the recognition and management of reduced kidney function in the clinical setting.

## Data

The dataset relied on in this article is available from the corresponding author on reasonable request.
